# The role of pyroptosis in heart failure and related traditional chinese medicine treatments

**DOI:** 10.3389/fphar.2024.1377359

**Published:** 2024-05-28

**Authors:** Jie Qin, Qianhe Yang, Yan Wang, Mengdi Shi, Xin Zhao, Yabin Zhou

**Affiliations:** ^1^ Graduate School, Heilongjiang University of Chinese Medicine, Harbin, Heilongjiang, China; ^2^ Department of Cardiovascular Medicine, First Affiliated Hospital of Heilongjiang University of Chinese Medicine, Harbin, Heilongjiang, China

**Keywords:** pyroptosis, the NLRP3 inflammasome, heart failure, mechanism, plant metabolites, botanical drug preparation

## Abstract

Pyroptosis is a type of programmed cell death that is mediated by both typical and atypical pathways and ultimately leads to the lysis and rupture of cell membranes and the release of proinflammatory factors, triggering an intense inflammatory response. Heart failure (HF) is a serious and terminal stage of various heart diseases. Myocardial hypertrophy, myocardial fibrosis, ventricular remodeling, oxidative stress, the inflammatory response and cardiomyocyte ionic disorders caused by various cardiac diseases are all risk factors for and aggravate HF. Numerous studies have shown that pyroptosis can induce and exacerbate these reactions, causing progression to HF. Therefore, targeting pyroptosis is a promising strategy to treat HF. This paper summarizes the role of pyroptosis in the development of HF and the underlying mechanism involved. Recent research progress on the ability of traditional Chinese medicine (TCM) extracts and formulas to inhibit pyroptosis and treat HF was summarized, and some traditional Chinese medicine extracts and formulas can alleviate different types of HF, including heart failure with preserved ejection fraction (HFpEF), heart failure with reduced ejection fraction (HFrEF), and heart failure with midrange ejection fraction (HFmrEF), by targeting pyroptosis. These findings may provide new ideas and evidence for the treatment or adjuvant treatment of HF by targeting pyroptosis.

## 1 Introduction

Heart failure (HF) is one of the most common cardiovascular diseases in the clinic and is the ultimate outcome of a variety of cardiovascular diseases. Weakened heart function due to ventricular filling and ejection dysfunction has become a serious public health problem due to its associated morbidity and mortality, and it places economic burdens on the healthcare system. The pathogenesis of HF is believed to be related to apoptosis, the inflammatory response, oxidative stress, cardiomyocyte fibrosis, and mitochondrial damage, and the inflammatory response is critical for the development of HF. The AHA Scientific Statement on Air Pollution and Cardiovascular Disease states that inflammatory mediators such as TNF-alpha and IL-1 cause systemic inflammation, which in turn damages the cardiovascular system. It has been demonstrated in animals and humans that the host can clear infected cells and externally invading pathogenic microorganisms through cell death (Galluzzi et al., 2018). Cell death regulates the dynamic equilibrium between physiology and pathology and plays a significant role in the development and maturation of organisms (Fuchs and Steller, 2011; Galluzzi et al., 2016a; 2016b; Jorgensen et al., 2017; Nagata and Tanaka, 2018). In recent years, pyroptosis, which is a newly discovered form of inflammatory programmed cell death, has been shown to be critical for the development of HF. In-depth study of the specific mechanism by which pyroptosis is involved in the occurrence and development of HF is critical for the discovery of novel targeted therapies and biomarkers for HF.

## 2 Pyroptosis in modern medicine

Many studies have confirmed that inflammatory reactions can cause myocardial cell damage under pathological conditions, leading to impaired cardiac function. Different types of cells in heart tissue interact to promote ventricular remodeling and the development of HF (Mauro et al., 2019; Zhang et al., 2019; Wu et al., 2021). Pyroptosis is a type of programmed cell death associated with the inflammatory response that was discovered in recent years. Pyroptosis is characterized by simultaneous apoptosis and necrosis, including nuclear condensation, organelle swelling, DNA breakage, cell membrane pore formation, and destruction of the cell membrane. The release of proinflammatory factors, including IL-1β, IL-18, high mobility group box-1 protein (HMGB-1) and heat shock protein (HSP), and other substances into the extracellular space triggers a series of inflammatory reactions, which can further cause organ dysfunction and promote the occurrence of disease (Bujak and Frangogiannis, 2009; Calvillo et al., 2011; Galluzzi et al., 2012; Yu et al., 2021).

Pyroptosis and apoptosis differ in that pyroptosis is not mediated by caspase-3 but involves inflammatory cysteine proteases. Researchers have identified inflammatory cysteine proteases, including Caspase-1/4/5, in humans as well as both classic and nonclassic pyroptosis pathways. The classic pathway depends on the activation of caspase-1. Pattern recognition receptors (PRRs) in cells recognize endogenous or exogenous stimuli. Inactive pro-caspase-1 indirectly binds to NOD-like receptors (NLRs) (NLRP1, NLRP3, NLRC4, and NLRC5) through the adaptor protein apoptosis-associated speck-like protein containing a CARD (ASC), which forms a high-molecular-weight protein complex called the inflammasome. These inflammatory vesicles are typically composed of proteins with a nucleotide binding oligomerization domain (NACHT), pyrin domain (PYD), and leucine-rich repeats (LRRs), caspase-1 precursor and ASC (von Moltke et al., 2013). Procaspase-1 is converted into active caspase-1 by the inflammasome. Caspase-1 can cause cell membrane perforation, leading to cell dissolution and death. The contents of cells are released into the extracellular space through the pores in the cell membrane, causing an inflammatory reaction. On the other hand, Caspase-1 can also activate IL-1β and IL-18 and cause them to be released from the cell. IL-1β and IL-18 trigger additional inflammatory cell accumulation, exacerbating the inflammatory response. Researchers have also shown that NLRP1 and NLRP4 can directly induce inflammatory reactions independent of caspase-1 (Van Opdenbosch et al., 2014). In the nonclassic pathway, LPS is mainly transported to the cytoplasm through the induction of Toll-like receptor-4 (TLR4), receptor for advanced glycosylation end products (RAGE), or outer membrane vesicles (OMVs). LPS can directly bind to caspase-4/5/11, resulting in incomplete cytosolic dysfunction and an inflammatory response after efflux of intracellular material, and caspase-4/5/11 can also exacerbate the inflammatory response to NLRP3 by inducing the outflow of potassium ions. Kayagaki et al. proposed that the protein gasdermin may be a downstream target of Caspase-1 and may be involved in pyroptosis (Kayagaki et al., 2015). Related studies have also suggested that the activation of GSDMD by caspase-8, neutrophil elastase, tissue protease G, the SpeB protease of group A *Streptococcus*, caspase-1/3/6/7, and granzyme A directly or indirectly leads to GSDMD cleavage or the specific cleavage of other factors to drive pyroptosis (Sarhan et al., 2018; Zhou et al., 2020; Deng et al., 2022; Wang and Ruan, 2022).

**FIGURE 1:**
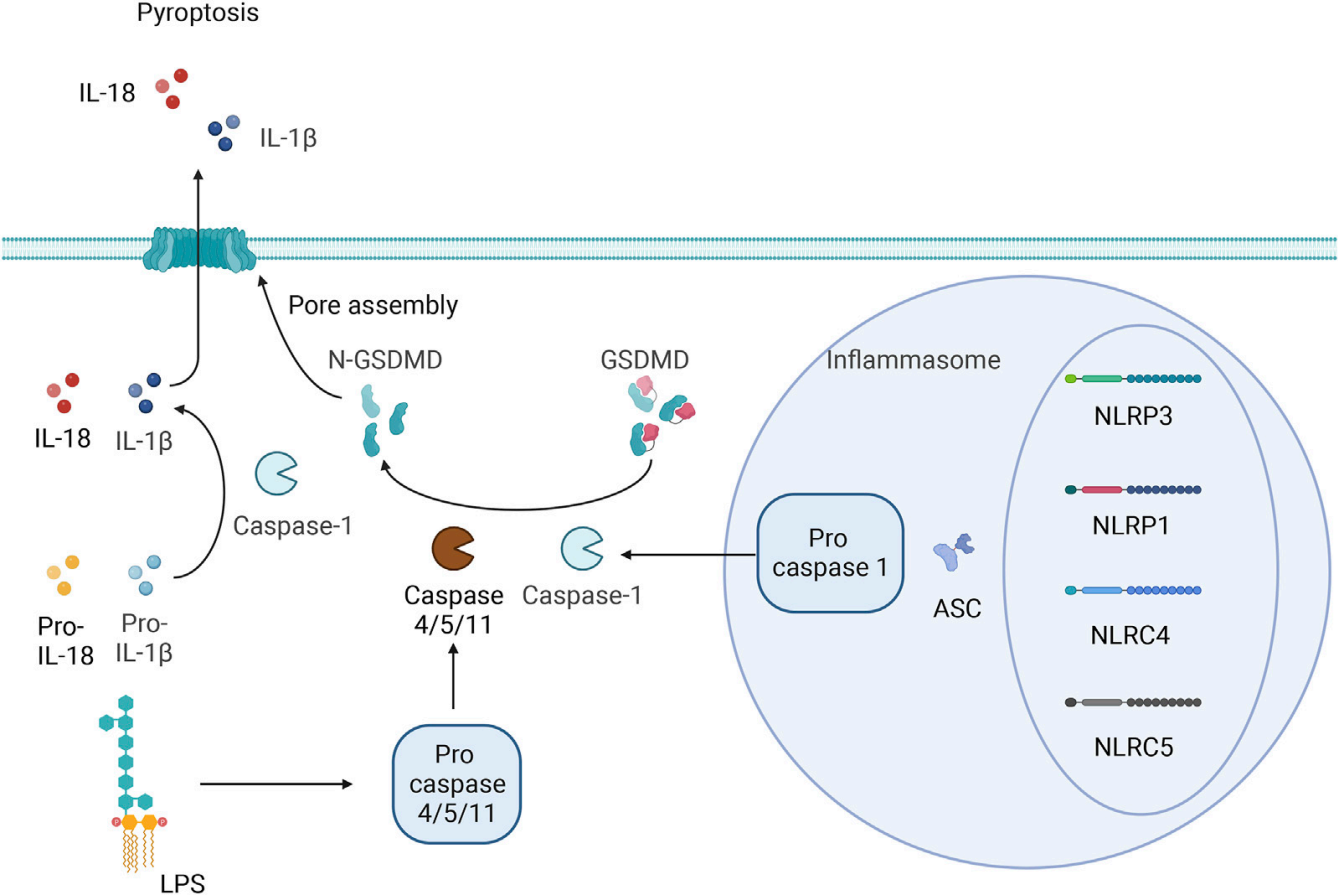
Classic and nonclassic pyroptosis pathways.

Caspase-1 cleaves the N- and C-terminal domains of GSDMD, resulting in the release of the N-terminal domain to mediate the formation of pyroptotic pores in the cell membrane; this causes the release of inflammatory factors to amplify inflammatory responses and induce pyroptosis (Hou et al., 2021). Jiang et al. (2022) suggested that cardiac function can be improved by inhibiting GSDMD cleavage; thus, GSDMD could be a new target for the treatment of HF. The specific details of the pyroptosis process are shown in Figure 1.

## 3 The role of pyroptosis in heart failure

### 3.1 Inflammation and pyroptosis in heart failure patients

Pathogens (pathogen-related molecular patterns [PAMPs]) or endogenous substances released during myocardial injury caused by ischemia or pressure overload (damage-related molecular patterns [DAMPs]) can be recognized by and activate PRRs in the heart (Mackey and McFall 2006; Mann, 2011). Important PRRs in the heart include NLRP3, Toll-like receptors (TLRs), and NLRs (Mann, 2011; Frantz et al., 2018; Toldo and Abbate, 2018). The activation of PRRs initiates downstream expression of the proinflammatory cytokines IL-1β, IL-18, TNF, and IL-6 and chemokines, leading to inflammatory reactions and inflammation-related pyroptosis. Although this innate immune system-induced inflammatory response has cytoprotective effects, such as via the clearance of necrotic cells and promotion of extracellular matrix degradation, when this inflammatory response is dysregulated, chronic inflammation can occur, leading to tissue damage that can result in left ventricular dysfunction and ventricular remodeling, ultimately leading to HF. During ischemic or nonischemic HF, chronic myocardial inflammation persists, which accompanies the progression of HF and leads to ongoing ventricular remodeling and the exacerbation of HF (Adamo et al., 2020). Persistent myocardial injury, activation of the sympathetic nervous system and the renin-angiotensin-aldosterone system, and hemodynamic overload during HF can all contribute to ongoing myocardial inflammation in HF patients (Gullestad et al., 1999; Torre-Amione et al., 1999; Mann, 2002). The proinflammatory cytokines IL-1β and IL-18, which are closely related to pyroptosis, have been proven to have negative contractile effects on the heart both *in vivo* in animals and *in vitro* (Gulick et al., 1989; Yokoyama et al., 1993; Yu et al., 2003; Toldo et al., 2014). The chronic inflammatory response in the myocardium can also reduce the expression ratio of matrix metalloproteinases (MMPs) to tissue inhibitors of metalloproteinases (TIMPs), thereby impairing the degradation of collagen deposited in the myocardium. The excessive deposition of collagen exacerbates the development of myocardial fibrosis and further exacerbates ventricular remodeling and HF (Zhang et al., 2011). In addition to the noncellular components mentioned above, the immune response related to chronic heart failure (CHF) also involves cellular components such as macrophages, mast cells, B cells, T cells, natural killer (NK) cells, and dendritic cells, which participate in sustaining chronic inflammation and ultimately lead to the exacerbation of HF (Noutsias et al., 2002; Bajpai et al., 2018).

### 3.2 Pyroptosis and myocardial fibrosis

**FIGURE 2:**
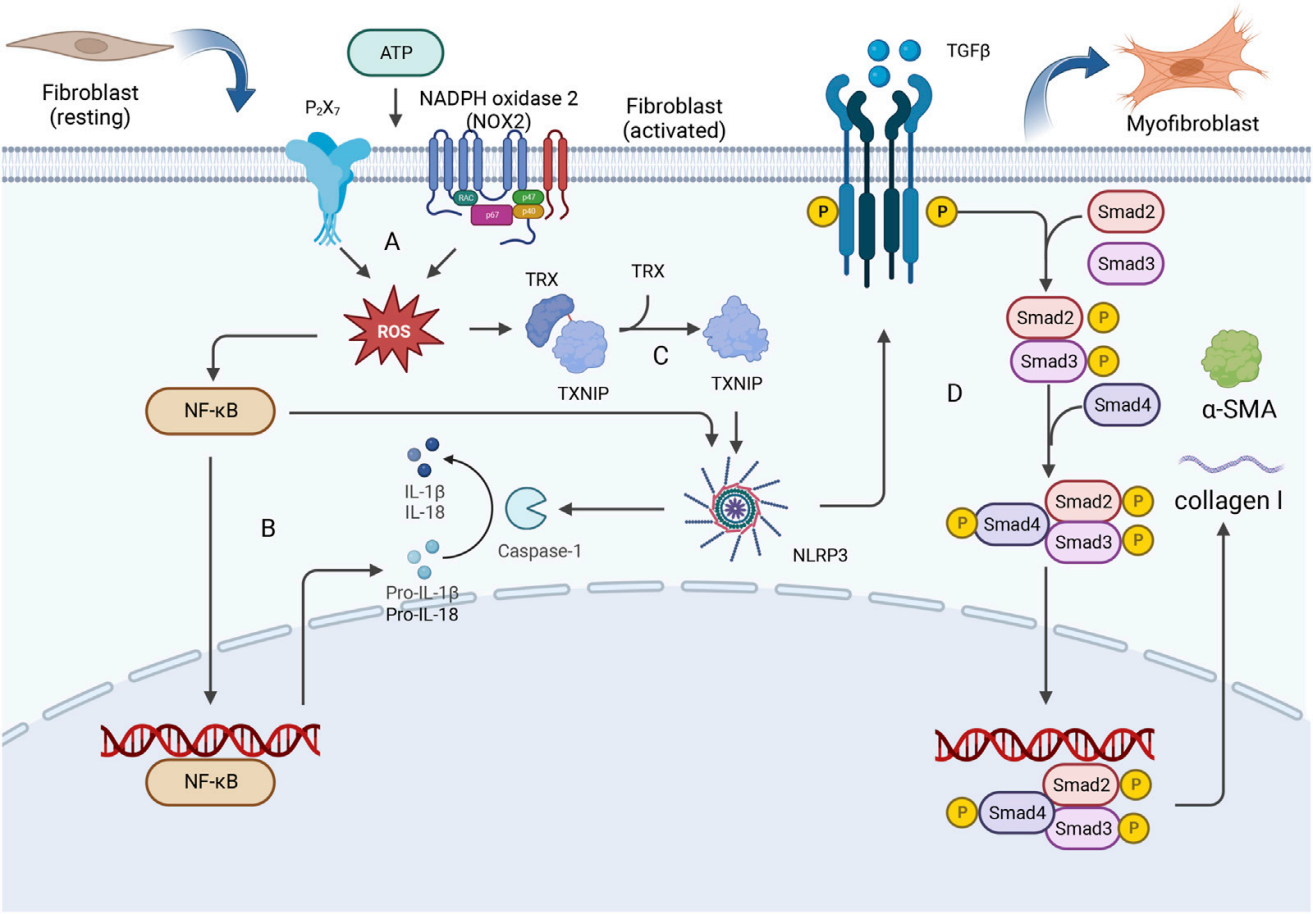
CFs are activated to form myofibroblasts (MFs) by a series of reactions involving NLRP3, leading to the development of myocardial fibrosis. **(A)** Extracellular ATP can stimulate P2X7 and NOX2 receptors, thereby mediating the production of intracellular ROS. **(B)** NF-κB is upregulated by ROS, and this upregulation promotes the expression of NLRP3 as well as the expression of pro-caspase-18 and pro-caspase-1β. **(C)** ROS stimulation induces the release of TXNIP from oxidized TRX, and TXNIP binds to the leucine-rich region of NLRP3, leading to inflammasome assembly. **(D)** The activation of NLRP3 triggers pyroptosis and the enhancement of the inflammatory response, thereby activating the profibrotic TGF-β/Smad signaling pathway. The Smad2-Smad3-Smad4 complex enters the nucleus, where it promotes the expression of collagen I and α-smooth muscle actin (α-SMA), leading to excessive deposition of type I collagen and ultimately resulting in myocardial fibrosis.

Myocardial fibrosis results from the excessive accumulation of extracellular matrix components in the myocardium and mostly involves cardiac fibroblasts. Previous studies have shown that myocardial ischemia‒reperfusion injury induces the production of ROS in cardiac fibroblasts and promotes the efflux of potassium ions, thus mediating the activation of the inflammasome in cardiac fibroblasts (Kawaguchi et al., 2011). ROS regulate the activation of the NLRP3 inflammasome by activating NF-κB, leading to potassium efflux and directly increasing the expression of NIMA-related kinase 7 (NEK7), which results in the formation of the NLRP3 inflammatory complex (He et al., 2016). Diabetic cardiomyopathy can initiate structural diseases (Santiago et al., 2014; Somanna et al., 2015). High blood glucose levels induce the generation of ROS during diabetic cardiomyopathy, which in turn upregulates NF-κB, and the upregulation of NF-κB promotes high expression of NLRP3, as well as the expression of pro-caspase-18 and pro-caspase-1β, stimulating the assembly of the inflammasome (Donath et al., 2010; Qiao et al., 2012; Boaru et al., 2015). Moreover, thioredoxin interacting/inhibiting protein (TXNIP) is expressed in response to ROS, which induces the assembly of NLRP3 inflammatory vesicles and regulates their structure (Martinon, 2010). In addition, attention should be paid to the potential development of lipotoxicity in patients with diabetes, and free fatty acids in the blood can also induce ROS production and ER stress, which in turn activate the NLRP3 inflammasome (Legrand-Poels et al., 2014). NLRP3 activation triggers the classic pyroptosis pathway and induces the production and release of proinflammatory cytokines, which in turn activate other immune cells and further enhance the inflammatory response. A variety of mechanisms cooperate to activate the profibrotic TGF-β/Smad signaling pathway (Henderson et al., 2020) and promote tissue fibrosis. The formation of mature IL-1β and IL-18 is catalyzed by the NLRP3 inflammasome, which promotes Ca2+ efflux from the sarcoplasmic reticulum and induces myocardial interstitial fibrosis. This disruption of tissue structure has an impact on the function of the heart and ultimately leads to HF (Zhang et al., 2021). CFs are activated to form myofibroblasts (MFs) by a series of reactions involving NLRP3, leading to the development of myocardial fibrosis. The specific details of this process are shown in Figure 2.

### 3.3 Pyroptosis and cardiac hypertrophy

Pathological myocardial hypertrophy is a key risk factor for HF (Zhang et al., 2021). Pressure overload can stimulate pathological hypertrophy in the myocardium. Studies have shown that pressure overload can induce pyroptosis in cardiomyocytes and that a caspase-1 inhibitor can inhibit pyroptosis. Moreover, this inhibitor was found to suppress the increase in heart weight and average myocardial cell surface area in the TAC mouse model, suggesting that the activation of pyroptosis is related to cardiac hypertrophy. This inhibitory effect was also experimentally shown to attenuate overload-induced HF, indicating that pyroptosis is closely related to the development of cardiac hypertrophy and HF (Yue et al., 2021). Mechanistically, NOX1 and NOX4 promote DrP1-mediated mitochondrial fission in the cardiomyocytes of patients with dilated cardiomyopathy (DCM), resulting in the accumulation of mitochondrial ROS, which activatess the NLRP3 inflammasome to induce pyroptosis. Therefore, pyroptosis is prevalent in the cardiomyocytes of patients with DCM and is important for the progression of DCM. A negative correlation between the left ventricular ejection fraction and cardiomyocyte death was also found in patients with DCM, suggesting that pyroptosis can lead to a progressive decline in cardiac function and ultimately to HF in these patients (Zeng et al., 2020). The late glycosylation end product receptor RAGE induces cardiac hypertrophy through activation of the PKC-ERK1/2 and NF-κB-NLRP3-IL1β signaling pathways, suggesting that RAGE-NLRP3 may be an important mediator of Ang II-induced cardiomyocyte hypertrophy (Lim et al., 2018). PKC is the main regulatory molecule that mediates compensatory cardiac hypertrophy (Wolny et al., 1997). When stimulated and activated, PKC can activate raf-1 and subsequently the MARK pathway; ERK1/2 is activated and phosphorylated and translocates to the nucleus via the action of raf proteins. In the nucleus, ERK can mediate a series of reactions, such as NF-κB activation, and the activation and nuclear translocation of NF-κB mediate the activation of many inflammatory factors and can cause pyroptosis, affecting cardiac function and ultimately leading to HF (Li and Guo, 2014).

### 3.4 Pyroptosis and oxidative stress

Activation of the sympathetic nervous system and the renin-angiotensin-aldosterone system are characteristic of HF. This activation process is related to oxidative stress in cardiac muscle cells. When there is an imbalance in oxidation‒reduction in cardiac muscle cells, the balance between the production and clearance of ROS is disrupted, affecting the antioxidant system in response to oxidative stress. As important products of oxidative stress, ROS have substantial impacts on the development of HF. With the onset of oxidative stress, high levels of ROS can lead to an increase in the diastolic stiffness of the left ventricle, which affects ventricular diastolic function and leads to unfavorable cardiac remodeling by inducing hypertrophic signaling, apoptosis and necrosis (Zhazykbayeva et al., 2020). Moreover, oxidative stress is associated with activation of the NLRP3 inflammasome. When inflammasome activators are stimulated, NLRP3 and ASC, which are typically located in the cytoplasm and endoplasmic reticulum, translocate to the mitochondrial-associated membrane (MAM), which affects MAM-mediated production of ROS (Zhao et al., 2022). The ability of targeted antioxidants to inhibit mitochondrial oxidative stress also verified that ROS are involved in pyroptosis. The mechanism involves molecules such as MFN2, VDAC, and mitochondrial antiviral signaling protein (MANS) located on MAMs, which mediate the production of ROS and activate the NLRP3 inflammasome. One experiment verified the dependency of NLRP3 activation on ROS produced by mitochondria by showing that an NLRP3 activator could not induce the production of IL-1β or activation of the inflammatory factor caspase-1 in VDAC-deficient cells (Sorbara and Girardin, 2011). Under endoplasmic reticulum stress (ERS) conditions, NOX4 activates NF-κB through ROS, thereby activating NLRP3 (Feng C. et al., 2017). Moreover, NOX2 regulates the expression of dsDNA-activated protein kinase R (PKR) by mediating PKR autophosphorylation, which mediates the assembly of the NLRP3 inflammasome (Li et al., 2010). The activation of NLRP3 and the formation of inflammasomes in cardiac muscle cells lead to an inflammatory response, and the release of proinflammatory cytokines can affect left ventricular function, exerting a negative inotropic effect, inhibiting myocardial contractility, inducing cardiac metabolic abnormalities, and promoting cardiac remodeling (Meldrum, 1998; Mann, 2002; Diwan et al., 2003). Oxidative stress, ROS, and NLRP3 promote the occurrence and development of HF.

### 3.5 Pyroptosis and ionic disorders

**FIGURE 3:**
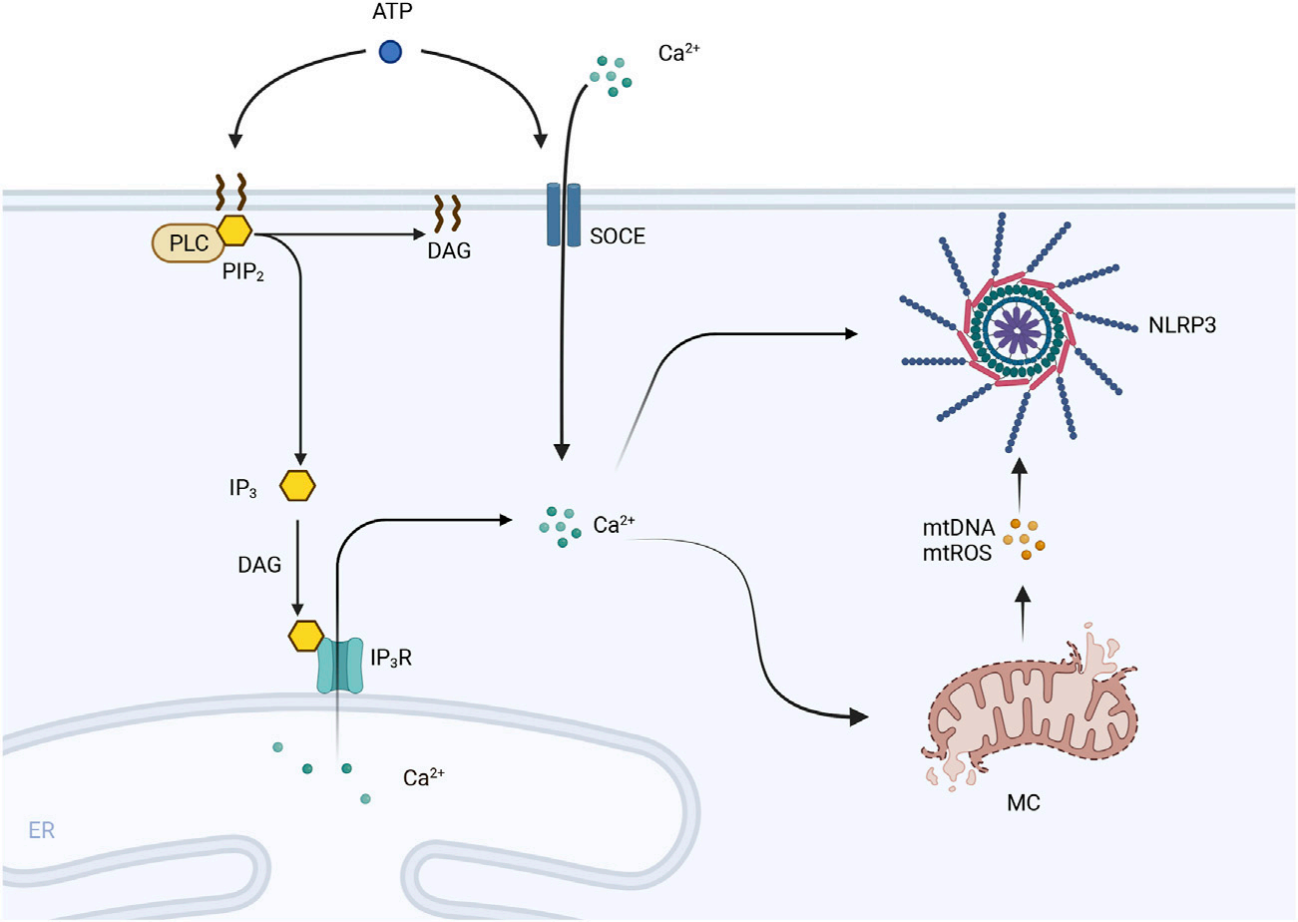
PLC is activated by extracellular ATP and hydrolyzes PIP2 in the cell membrane into DAG and IP3. IP3 is induced to interact with the IP3 receptor (IP3R) on the endoplasmic reticulum (ER) by DAG, leading to the efflux of Ca2+ stored in the ER into the cytoplasm. Moreover, SOCE channels are activated by extracellular ATP, and extracellular Ca2+ flows inward, leading to an increase in the intracellular cytoplasmic Ca2+ concentration. Excessive Ca2+ uptake leads to mitochondrial damage, including mitochondrial pore opening and rupture. The contents of mitochondria, including mtDNA and mtROS, are released and activate the NLRP3 inflammasome.

In a normal heart, the same amount of Ca2+ ions leave cardiomyocytes as enter them to maintain homeostasis. In contrast, Ca2+ release, uptake and efflux are impaired under pathological conditions or due to disruption of the T-tubule network and abnormalities in sarcoplasmic reticulum Ca2+ release channels (Ahmmed et al., 2000), and the abnormal release of Ca2+ from the sarcoplasmic reticulum has been shown to promote centrifugal myocardial remodeling and pumping deficits and ultimately lead to HF (Sedej et al., 2014). Impaired myocardial Ca2+ homeostasis affects cardiac contractile function and can lead to structural remodeling, which is a key factor in the development of HF. Moreover, experimental studies have shown that Ca2+ mobilization can regulate activation of the NLRP3 inflammasome by affecting the upstream and proximal steps of NLRP3 inflammasome activation. Phospholipase C protein (PLC), the IP3 receptor, and store-operated Ca2+ entry (SOCE) are activated by extracellular ATP stimulation and cause the release of Ca2+ from the endoplasmic reticulum and the influx of extracellular Ca2+. These findings indicate that both factors are necessary for the activation of the NLRP3 inflammasome, while other studies have revealed that inhibitors of PLC, the IP3 receptor, and SOCE inhibit ATP-stimulated Ca2+ flux and block caspase-1 and IL-1β processing (Murakami et al., 2012). Excess Ca2+ uptake can damage mitochondria and cause the generation of large amounts of ROS, which in turn open the mitochondrial pore and rupture mitochondria, from which mtDNA, oxidized mtDNA, and mtROS are released; these three factors have a damaging effect that can activate the NLRP3 inflammasome (Murakami et al., 2012; Zhazykbayeva et al., 2020). The specific details of this process are shown in Figure 3.

### 3.6 Pyroptosis and HF

The classic pyroptosis pathway includes NLRP3, NLPR1, NLRC4 and AIM2. Low-level basal activation of the NLRP3 inflammasome contributes to the progression of HF (Toldo et al., 2022). The activation of AIM2 and the NLRC4 inflammasome is also closely related to chronic inflammation in HF (Onodi et al., 2021). During HF, the inflammosome can directly sense pathological stimuli and be activated in cardiomyocytes and cardiac fibroblasts, causing pyroptosis and the release of proinflammatory factors, which in turn trigger cardiac inflammation and aggravate HF (Wu et al., 2021). Pyroptosis can also directly lead to the death of cardiomyocytes and reduce the number of functional cardiomyocytes, thereby affecting the systolic and diastolic function of the myocardium and promoting cardiac remodeling in patients with HF (Chai et al., 2022).

## 4 Treatment of HF with TCM formulations inhibits pyroptosis

TCM pharmacological studies mostly focus on the effects of individual metabolites and botanical drug preparations. Below, we review the research progress on therapeutic interventions for inhibiting pyroptosis in HF using botanical drug preparations comprising different plant metabolites.

### 4.1 Plant metabolites

#### 4.1.1 Puerarin

Puerarin is a metabolite of the botanical drug *Pueraria lobata* [Fabaceae; Puerariae lobatae radix] (Ge Gen). Some researchers have shown that it has anti-inflammatory and antioxidant effects and can reduce organ fibrosis (Zhang et al., 2006; Liu et al., 2013; Wang et al., 2016; Lin et al., 2017; Zhou et al., 2017). Through experiments on an heart failure with preserved ejection fraction (HFpEF) rat model, puerarin was shown to significantly improve cardiac function and reduce the levels of inflammatory markers such as TNF-α, IL-1β, and IL-6 in the serum. These findings indicate that puerarin can inhibit the persistent inflammatory response in HFpEF and improve myocardial function by regulating myocardial pyroptosis (He et al., 2019). Further studies have shown (Ni, 2022) that puerarin can inhibit PARP-1 and prevent the release of high mobility group protein β1, thereby inhibiting the TLR4-NF-κB pathway, reducing the release of proinflammatory factors, reducing the degrees of inflammation and myocardial fibrosis, and contributing to the alleviation of HFpEF.

#### 4.1.2 Resveratrol

Resveratrol is a natural polyphenolic metabolite that is mainly derived from the botanical drug Polygonum cuspidatum Sieb. et Zucc [Polygonaceae; Polygoni cuspidati rhizoma et radix] (Hu Zhang), Cassia obtusifolia L [Fabaceae; Cassiae semen] (Jue Ming Zi) and *Morus alba* L [Moraceae; Mori fructus] (Sang Shen) (Berretta et al., 2020). It has anti-inflammatory, antioxidant and antiapoptotic effects. In a pressure overload-induced heart failure with reduced ejection fraction (HFrEF) mouse model, resveratrol was shown to significantly increase survival, and subsequent experiments demonstrated that resveratrol improves diastolic function in mice with HFrEF. Resveratrol can also decrease the degree of myocardial fibrosis in mice with HFrEF, improve left ventricular diastolic filling, and reduce the size of the left ventricle in mice with HFrEF (Sung et al., 2015). In ischemic heart disease, resveratrol inhibits the accumulation of NF-κB/p65 in the nucleus in ischemic areas, thereby blocking the mRNA expression of inflammatory cytokines such as IL-1β, IL-16, and TNF-α. Resveratrol inhibits the NLRP3 inflammasome and inflammatory reactions in different cell types involved in ischemic heart disease and prevents the progression of ischemic heart disease to HFrEF (Feng et al., 2020). To reduce the risk of HFrEF, resveratrol can improve cardiac function and ameliorate left ventricular fibrosis after acute myocardial infarction by inhibiting the TGF-β1/SMAD2 signaling pathway and NLRP3 inflammasome activity (Jiang et al., 2021). Research has shown that with the development of coronary microembolization (CME), gradual cardiac failure and progressive myocardial damage can occur; this may be related to NLRP3-mediated pyroptosis and the inflammatory response. Resveratrol may alleviate CME-induced myocardial pyroptosis by inhibiting the TLR4/MyD88/NF-κB signaling pathway (Luo et al., 2023).

#### 4.1.3 Triptolide

Triptolide (TP), which is a metabolite of the botanical drug Tabellae tripterygii hypoglauci [Celastridae; Tripterygium wilfordii Hook.f.] (Lei Gong Teng), is a diterpenoid that has been reported to alleviate isoproterenol-induced cardiac hypertrophy in mice, myocardial fibrosis, and cardiac remodeling in mice (Liu et al., 2015; Ding et al., 2016). TP inhibits the activation of the NLRP3 inflammasome by inhibiting the NF-κB/NLRP3 pathway. TP decreases inflammation in myocardial fibrosis and reduces the mRNA expression of inflammatory factors such as IL-1β, TNF-α, IL-18, monocyte chemoattractant protein 1 (MCP-1) and vascular cell adhesion molecule 1 (VCAM-1), which in turn alleviates myocardial fibrosis and protects cardiac function (Shen et al., 2021). Pan et al. (Pan et al., 2019) performed a series of clinically relevant experiments and concluded that TP can inhibit the assembly of the NLRP3 inflammasome, thereby inhibiting AngII-induced activation of the NLRP3 inflammasome and the subsequent release of the inflammatory factors IL-1β and IL-18. It was also found to block the activation of the MAPK-TGFβ1-Smad signaling pathway, thereby inhibiting the generation of collagen in cardiac fibroblasts. *In vivo* experiments also confirmed that TP can alleviate cardiac fibrosis through this mechanism. However, the mechanism by which TP affects NLRP3 through various pathways needs to be further studied, and the targets and pathways of TP in HFrEF need to be determined.

#### 4.1.4 Ginsenoside Rg1

Ginsenoside Rg1 (GRg1) is a metabolite of the botanical drug Panax ginseng C. A. Mey [Araliaceae; Ginseng radix et Rhizoma] (Ren Shen) and has antiaging, anti-inflammatory, antiapoptotic and antioxidant effects (Luo et al., 2019; Liu et al., 2021). Zheng et al. (Zheng et al., 2017) reported that GRg1 can attenuate myocardial fibrosis in rats with HF by regulating fibrosis-associated proteins such as AngII, ACE, AT1, osteopontin, and collagen type I. GRg1 also has a similar effect as TP. It can inhibit the TGFβ1-Smad signaling pathway to attenuate myocardial fibrosis in rats with HF. GRg1 may also reduce HF-induced myocardial hypertrophy by inhibiting the ERK signaling pathway. Additionally, GRg1 inhibits the activation of the NLRP3 inflammasome in cardiac fibroblasts, which in turn inhibits the release of IL-1β and pyroptosis; blocks the activation of cardiac fibroblasts, the synthesis of collagen and the accumulation of ECM proteins; mitigates the progression of myocardial fibrosis and associated damage to myocardial tissues and functions; and delays the onset and development of HFrEF. However, the mechanisms underlying these effects are not completely understood and need to be further studied in depth (Kan, 2023).

#### 4.1.5 Dihydromyricetin

Dihydromyricetin (DHM), also known as snake graphene, is a natural flavonoid that is abundant in Ampelopsis sinica (Miq.) W.T. Wang [Vitis Sinica Miq. ; *A. heterophylla* (Thunb.) Sieb. et Zucc. var. vestita Rehd] (She Pu Tao) and has anti-inflammatory, antithrombotic, antioxidant, and antitumor effects (Wang et al., 2018). Activation of the inflammatory response by the NLRP3 inflammasome may be one of the mechanisms by which adriamycin induces myocardial injury and HFpEF, and this finding may lead to new treatment ideas and methods. By examining adriamycin (DOX)-induced HFpEF in rats, Sun et al. (Sun et al., 2020) found that DHM inhibits DOX-induced activation of the NLRP3 inflammasome by activating the SIRT1 pathway and suppressing DOX-induced increases in IL-1β and IL-18 levels in plasma, alleviating DOX-induced cardiomyocyte apoptosis and left ventricular dysfunction.

#### 4.1.6 Colchicine

Colchicine is a zhuophenone alkaloid extracted from Iphigenia indica Kunthet Benth [Liliaceae; Iphigenia indica kunth corm] (Lijiang Shan Ci Gu). In recent years, colchicine has mostly been used for the treatment of gout, and subsequent studies have shown that it can be used for the treatment of a wide range of inflammatory conditions. As research on inflammation has increased, the pathological role of inflammation in a variety of diseases has been revealed. Numerous studies have shown that colchicine may ameliorate and prevent HF by inhibiting the inflammatory response. Colchicine improves cardiac function and slows the development of cardiac fibrosis in a rat model of HFpEF, possibly by inhibiting the activation of the NLRP3 inflammasome and the NF-κB pathway and attenuating the inflammatory response associated with pyroptosis (Song et al., 2022). Colchicine interferes with the assembly of the NLRP3 inflammasome in the damaged myocardium of dilated cardiomyopathy patients by enhancing the expression of SIRT2 to promote the deacetylation of NLRP3, which in turn mitigates further exacerbation of dilated cardiomyopathy, suggesting the possibility of targeting pyroptosis for the treatment of CHF (Sun et al., 2022). Colchicine inhibits the expression of NLRP3 mRNA in the infarct zone after myocardial infarction and reduces the levels of M1 inflammatory cytokines, such as TNF-α, IL-1β, and IL-6, in the infarct zone, ameliorating HFpEF after myocardial infarction. This may be the key mechanism by which colchicine attenuates adverse ventricular remodeling and ventricular dysfunction in the chronic phase of myocardial infarction (Koichiro et al., 2017).

#### 4.1.7 Tanshinone IIA

Tanshinone IIA is a lipophilic diterpene isolated from Salvia miltiorrhiza Bunge [Lamiaceae; Salviae miltiorrhizae radix et rhizoma] (Dan Shen). Modern pharmacological studies have shown that tanshinone IIA has anti-inflammatory and antioxidant activities and has beneficial effects in cardiovascular diseases (Guo et al., 2020). Tanshinone IIA downregulates the expression of TLR4 and NF-κB, inhibiting the nuclear translocation of NF-κB p65 in the cardiomyocytes of acute myocardial infarction-induced HFpEF model rats. This finding suggests that tanshinone IIA may improve cardiac function and reduce myocardial injury in HFpEF by inhibiting the TLR4/NF-κB p65 signaling pathway and the related inflammatory response and subsequently inhibiting cardiomyocyte pyroptosis in model rats (Chai et al., 2023). Coronary microembolization (CME) can lead to myocardial injury and a decrease in cardiac function and is a reliable predictor of serious cardiac events. Myocardial pyroptosis was found to be involved in the development of CME; however, tanshinone IIA can delay this process. The underlying mechanism is that tanshinone IIA reduces cardiomyocyte pyroptosis by inhibiting the TLR4/MyD88/NF-κB/NLRP3 pathway (Li et al., 2022). The mechanisms by which plant metabolites inhibit pyroptosis are listed in Table 1. Details of the plant metabolites involved can be found in (Supplementary Data Sheet 1.ZIP)

**TABLE 1:** Mechanisms by which plant metabolites inhibit pyroptosis.

Herbal extracts	Plant	Type of extract	Range tested	Model			Control				Treatment time		Mechanism and effect	Reference
							*In vivo*		*In vitro*					
					*In vivo*	*In vitro*	Control group	Experimental group	Control group	Experimental group	*In vivo*	*In vitro*		
Puerarin	Pueraria lobata	Isoflavones	400 mg/kg/d	The rat model of cardiac fibrosis was established by abdominal aortic coarctation	Male Wistar rats	Rat cardiac fibroblasts	Sham group; TAC group	TAC + puerarin group	Control group; LPS group	LPS + Puerarin group; LPS + Puerarin + PARP-1 group; LPS + Puerarin + PARP-1+siHMGB1 group	10 weeks	LPS:12h; Puerarin:3h; PARP1:4–6h; siHMGB1:4–6 h	Down-regulating PARP-1 inhibits the HMGB1-mediated NF-kB proinflammatory pathway, alleviates the inflammatory cascade, and has a protective effect on cardiac fibrosi	Ni. (2022)
			60 mg/kg/d	Ligation of the left anterior descending (LAD)establish coronary arterychronic heart failure model	Sprague-Dawley (SD) rats	Rat myocardial cells	Sham group; CHF model + PBS group	CHF model + puerarin group	PBS control group; siRNA-negative control group; si-PPARα group	Puerarin group; si-PPARα+puerarin group	4 weeks	PBS:12h; Puerarin:12h; siRNA-negative control:72h; si-PPARα:72 h	The apoptosis and inflammatory response of CHF rat cardiomyocytes may be inhibited by PPARα pathway	He et al. (2019)
Resveratrol	Linum usitatissimum, Polygonum cuspidatum, Cassia seed, Mulberry	Polyphenolic compound	320 mg/kg/d	C57Bl/6 mice were subjected to either sham or transverse aortic constriction surgery to induce HF	Male C57B1/6 mice		Sham group; TAC-HF group	TAC-HF + Resveratrol group			2 weeks		Decrease the degree of myocardial fibrosis in mice with HF, improve left ventricular diastolic filling properties, and reduce the size of the left ventricle in mice with HF	Sung et al. (2015)
			320 mg/kg/d	Classic permanent coronary occlusion without reperfusion was performed to induce myocardial ischemia and myocardial infarction (MI) according to published methods	Male mice (C57BL/6J)	Neonatal rat cardiomyocytes (NRCMs) and cardiac fibroblasts (CFs)	Normal saline (NS) group; MI + NS group	Resveratrol group; MI + resveratrol group	Lipopolysaccharide (LPS) group	LPS + resveratrol group	3 weeks	LPS:12h; Resveratrol:12 h	Treatment with Resveratrol blocks the accumulation of NF-κB/p65 in the nucleus of the ischemic region, thereby blocking the expression of inflammatory cytokine mRNA. Resveratrol treatment inhibited the NLRP3 inflammatome and associated inflammatory response in different cell types of ischemic heart disease, preventing the further development of ischemic heart disease to heart failure	Feng et al. (2020)
			50 mg/kg/d	The rat AMI model was established by the permanent ligation of left anterior descending coronary artery method	Male Sprague-Dawley (SD) rats		Sham group; AMI group	Sham + resveratrol; AMI + resveratrol			45 days		Resveratrol can improve cardiac function and left ventricular fibrosis after acute myocardial infarction and reduce the risk of HF by inhibiting TGF-β1/SMAD2 signaling pathway and NLRP3 inflammatome activity	Jiang et al. (2021)
			25 mg/kg/d; 50 mg/kg/d	A thoracotomy was then carried out at the left margin of the sternum in the fourth and third intercostal spaces. The ascending aorta was then entirely separated and exposed before being clamped for about 10 s implementing a vascular clamp. An injection comprising three thousand 42 μm diameter polyethylene microspheres suspended within physiological saline (0.1 mL) was given into the rat cardiac apex in the model groups simultaneously but fast	Male Sprague-Dawley (SD) rats		Sham group; CME group	CME + RES (25 mg/kg); CME + RES (50 mg/kg)			7 days		Resveratrol alleviates CME-induced myocardial pyrodeath by inhibiting TLR4/MyD88/NF-κB signaling pathway	Luo et al. (2023)
Triptolide	Thunder god vine	Diterpenoid ingredient	10 μg/kg; 30 μg/kg; 100 μg/kg	Isoproterenol-Induced Cardiac Fibrosis in Mice	Adult male C57 mice	Mouse cardiac fibroblasts (CFs)	Control group; No TP + Iso group	TP (100 μg/kg) group; ISO + TP (10 μg/kg) group; ISO + TP (30 μg/kg) group; ISO + TP (100 μg/kg) group	Wildtype CFs medium; NLRP3-knoclcont CFs medium; Flag-NLRP3 CFs medium	Wildtype CFs + AngII; Wildtype CFs + TP; Wildtype CFs + AngII + TP; NLRP3-knoclcont CFs + AngII; NLRP3-knoclcont CFs + TP; NLRP3-knoclcont CFs + AngII + TP; Flag-NLRP3 CFs + AngII; Flag-NLRP3 CFs + TP; Flag-NLRP3 CFs + AngII + TP	14 days	AngII:24h; TP:24 h	Inhibiting the assembly of the NLRP3 inflammasome inhibits activation of the NLRP3 inflammasome by AngII and the subsequent release of inflammatory factors IL-1β and IL-18, blocking activation of the MAPK-TGFβ1-Smad signaling pathway and inhibiting the production of collagen in cardiac fibroblasts	Pan et al. (2019)
			200 μg/kg/d	Iso-induced myocardial fibrosis in rats	Adult male Sprague-Dawley (SD) rats		Sham group; MF group	Dimethyl sulfoxide (DMSO) group; TPL group			7 days		Triptolide inhibits the activation of the NLRP3 inflammasome by inhibiting the NF-κB/NLRP3 pathway. Triptolide can downregulate the inflammatory level of myocardial fibrosis and reduce the mRNA expression of inflammatory factors IL-1β, TNF-α, IL-18, MCP-1 and VCAM-1, thereby improving the degree of myocardial fibrosis and protecting cardiac function	Shen et al. (2021)
Ginsenoside Rg1	Ginseng	Triterpenoid saponins	50 mg/kg/d	Mice myocardial fibrosis model was constructed by ISO	Adult male C57BL/6 mice	Primary cardiac fibroblasts from neonatal C57BL/6 mice	Control group; ISO group	Ginsenoside Rg1 treatment group	Control group; LPS Model group	Ginsenoside Rg1 intervention group; NLRP3 inhibitor (MCC950) group	14 days	LPS:6h; Ginsenoside Rg1:7h; MCC950:7 h	It downregulates NLRP 3/Caspase-1 signaling pathway, inhibits CF pyroptosis, and reduces CF activation and Co l-I synthesis	Kan (2023)
			35 mg/kg/d; 70 mg/kg/d	HF was induced by abdominal aortic coarctation	Male Sprague-Dawley (SD) rats		Sham group; heart failure group	Low dose of ginsenoside Rb1 group; high dose of ginsenoside Rb1 group; Losartan group			8 weeks		GRg1 can attenuate myocardial fibrosis in rats with HF by regulating fibrosis-associated proteins such as AngII, ACE, AT1, osteopontin, and collagen type I. It can inhibit the TGFβ1-Smad signaling pathway to attenuate myocardial fibrosis in rats with HF.	Zheng et al. (2017)
Dihydromyricetin	Ampelopsis glandulosa Momiy	Flavonoids	100 mg/kg/d; 200 mg/kg/d	Dox-induced cardiomyopathy in rats	Sprague-Dawley (SD) rats	H9C2 cells	Control group; DOX group	DOX + DHM-100 group; DOX + DHM-200 group	Control group; DOX group	DHM group; DOX + DHM group	6 weeks	24 h	DHM inhibited DOX-induced activation of the NLRP3 inflammasome by activating the SIRT1 pathway, and suppressing DOX-induced increases in IL-1β and IL-18 in plasma, which alleviated cardiomyocyte apoptosis and left ventricular dysfunction in rats induced by DOX.	Sun et al. (2020)
Colchicine	The bulb of Colchicum autumnale	Alkaloid	0.1 mg/kg/d	The Dahl/SS rats were fed with a high salt feed (8% NaCl) for 6 weeks to generate the diastolic dysfunction model, which subsequently progressed to HFpEF.	Male Dahl salt-sensitive rats		High salt diet group; low salt diet group	High salt + colchicine group			11 weeks		Colchicine inhibits the activation of NLRP3 inflammasome and NF-κB pathway, alleviates associated inflammatory response and cell pyrodeath, and improves cardiac function in HFpEF rat models	Song et al. (2022)
			0.4 mg/kg/d	To establish chronic dilated cardiomyopathy model (DCM), mice were administered intraperitoneally with 5 mg/kg doxorubicin weekly for consecutive 4 weeks at a cumulative dose of 20 mg/kg	C57BL/6 male mice	Primary neutrophils were collected from 4-week-old mice bone marrow	Control group; DCM group	DCM + Colchicine group	Control group; Doxorubicin + Nig group	Doxorubicin + Nig + Colchicine; Doxorubicin + Nig + Colchicine + AGK2	4 weeks	4 h	Colchicine promoted the deacetylation of NLRP3 by enhancing the expression of SIRT2, and interfered with the assembly of NLRP3 inflammasome in damaged myocardium, thus alleviating the further deterioration of the contraindicated heart	Sun et al. (2022)
			0.1 mg/kg/d	MI was induced by permanent occlusion of the left anterior descending coronary artery	Male C57BL/6J mice		Sham-Vehicle; MI-Vehicle	Sham-Colchicine; MI-Colchicine			4 weeks		Colchicine inhibited the mRNA expression of NLRP3 components and decreased the M1 inflammatory cytokines in the infarct area after myocardial infarction. This may be the key mechanism by which Colchicine alleviates poor ventricular remodeling and ventricular dysfunction during chronic myocardial infarction, and improves HF after myocardial infarction	Koichiro et al. (2017)
Tanshinone IIA	The rhizome of Salvia miltiorrhiza	lipophilic diterpenes	1.5 mg/kg/d	A rat model of heart failure (HF) induced by acute myocardial infarction (AMI) was established via ligation of the left anterior descending coronary artery	Male Sprague-Dawley (SD) rats	H9C2 cells	Shan group; Model group	Tanshinone ⅡA group; Captopril group	Control group	Hypoxia/Reoxygenation (H/R) group; Tanshinone IIA group	8 weeks	24 h	Tanshinone IIA can downregulate the expression of TLR4 and NF-κB in HF rat cardiomyocytes induced by acute myocardial infarction, and inhibit the nuclear translocation of NF-κB p65	Chai et al. (2023)
			10 mg/kg/d; 20 mg/kg/d	The rat CME model:The ascending aorta was separated, followed by clamping for 10 s using a vascular clamp after a left lateral thoracotomy was completed via the 3rd and 4th intercostal gaps. In addition, 3,000 polyethylene microspheres with a diameter of 42 μm (Biosphere Medical Inc., Rockland, MA, USA) were suspended in 0.1 mL of normal saline, followed by rapidly introducing into the left ventricle with microinjector	Male Sprague-Dawley (SD) rats		Sham group; The CME group	The CME + low-dose Tan IIA group; The CME + high-dose Tan IIA group			1 week		Tanshinone IIA can reduce myocardial pyrodeath by inhibiting the TLR4/MyD88/NF-κB/NLRP3 pathway	Li et al. (2022)

### 4.2 Botanical drug preparation

#### 4.2.1 Shenfu injection

Shenfu injection is a botanical drug preparation composed of red Ren Shen and Aconitum carmichaelii Debx [Ranunculaceae; Aconiti lateralis radix praeparata] (Fu Zi) extracts and was developed from the famous traditional Chinese formula Shenfu Soup. Shenfu injection significantly increases the LVEF and LVFS in isoproterenol-induced rats with HFpEF and significantly reduces NT-proBNP levels. Observations of tissue sections from rats with HFpEF revealed that Shenfu injection alleviates pathological damage to cardiac tissues and reduces the fibrotic area of cardiomyocytes in rats with HFpEF. Further studies revealed that Shenfu injection decreases the expression levels of NLRP3, caspase-1, IL-1β, IL-18, and GSDMD, indicating that Shenfu injection inhibits the classic NLRP3 inflammasome pathway, which in turn inhibits cardiomyocyte pyroptosis and attenuats inflammatory responses in rats with HFpEF and delays the further development of HFpEF (Fan et al., 2023).

#### 4.2.2 Shenkui Tongmai granule

Shenkui Tongmai granule is a botanical drug preparation created based on the clinical experience of Mr. Zhou Zhongying, who is a master of Chinese medicine. It is mainly composed of the botanical drugs Huang Qi, Cinnamomum cassia Presl [Lauraceae; Cinnamomi ramulus] (Gui Zhi), Ganoderma lucidum (Leyss.ex Fr.) Karst [Polyporaceae; Ganoderma] (Ling Zhi), Epimedium brevicornu Maxim [Berberidaceae; Epimedii folium] (Yin Yang Huo), Dan Shen, Abelmoschus manihot (L.) Medic [Malvaceae; Abelmoschi corolla] (Huang Shu Kui Hua), Poria cocos (Schw.) Wolf [Polyporaceae; Poria] (Fu Ling) and Citrus reticulata Blanco [Rutaceae; Citri Reticulatae Pericium] (Chen Pi). Experiments on heart failure with midrange ejection fraction (HFmrEF) model rats revealed that Shenkui Tongmai granule significantly reduces the LVEDD, LVESD, and LVMI and significantly increases the LVEF. Further studies showed that the expression levels of NLRP3, caspase-1, IL-1β and IL-18 are reduced in HFmrEF model rats treated with Shenkui Tongmai granules, resulting in the inhibition of NLRP3 inflammasome activation, pyroptosis and subsequent inflammatory reactions. Moreover, the number of TUNEL-positive cardiomyocytes among HFmrEF model rat cardiomyocytes was found to be significantly reduced by Shenkui Tongmai granule treatment, while the release of the immunoinflammatory factors Caspase1 and IL-1β was shown to be reduced, which further proved that Shenkui Tongmai granules inhibit pyroptosis in HFmrEF model rat cardiomyocytes. Inhibiting TLR4 and NF-κB also reduces the activation of inflammatory factor precursors, which suppresses the inflammatory response during HFmrEF and effectively improves cardiac function and ameliorates myocardial remodeling (Yan et al., 2019).

#### 4.2.3 Shengui-Yixin soup

Shengui-yixin soup is derived from the classic formula Zhi Gan Cao soup reported in the Treatment of Febrile Diseases and from Huangqiguizhi Wuwu soup reported in the Synopsis of the Golden Chamber. Shengui-Yixin soup consists of Huang Qi, Pseudostellaria heterophylla (Miq.) Pax ex Pax et Hoffm [Caryophyllaceae; Pseudostellariae radix] (Tai Zi Shen), Glycyrrhiza uralensis Fisch [Fabaceae; Glycyrrhizae radix et rhizoma] (Gan Cao), Gui Zhi, Dan Shen, Descurainia sophia (L.) Webb. ex Prantl [Brassicaceae; Descurainiae semen lepidii semen] (Ting Li Zi), Alisma orientale (Sam.) Juzep [Alismataceae; Alismatis rhizoma] (Ze Xie), Carthamus tinctorius L [Asteraceae; Carthami flos] (Hong Hua), Lycopus lucidus Turcz. var. Hirtus Regel [Lamiaceae; Lycopi Herba] (Ze Lan), and Chen Pi. It was clinically observed (Feng, 2015) that Shengui-Yixin soup can ameliorate the clinical symptoms of patients with HFmrEF, enhance myocardial contractility, reduce cardiomyocyte damage and alleviate ventricular remodeling in patients. Further studies have shown that by inhibiting the expression of TLR4, Shengui-Yixin can block the binding of the downstream molecule MyD88 to the death domain of IL-1 receptor-associated kinase (IRAK) and trigger the phosphorylation of IRAK, which in turn inhibits the activation and translocation of NF-κB and reduces the production of the downstream inflammatory factors TNF-α, IL-6, and IL-1β, alleviating inflammatory responses in patients with HFmrEF and delaying the development of HFmrEF (Feng et al., 2017).

#### 4.2.4 Xinkang granule

Xinkang granule is made by modifying the prescriptions of Sheng Xian Tang, Zhen Wu Tang, and Shenling Baizhu San. It is composed of a variety of botanical drugs, such as Huang Qi, Ren Shen, Cimicifuga heracleifolia Kom [Ranunculaceae; Cimicifugae rhizoma] (Sheng Ma), Bupleurum chinense DC [Apiaceae; Bupleuri radix] (Chai Hu), Platycodon grandiflorum (Jacq.) A. DC [Campanulaceae; Platycodonis radix] (Jie Geng), Fu Ling, Chen Pi, Atractylodes macrocephala Koidz [Asteraceae; Atractylodis macrocephalae rhizoma] (Bai Zhu), Coix lacryma-jobi L. var.ma-yuen (Roman.) Stapf [Poaceae; Coicis semen] (Yi Yi Ren), Prunus armeniaca L. var. Gansu eva Maxim [Rosaceae; Armeniacae semen amarum] (Ku Xing Ren), Fu Zi, Gui Zhi, Zingiber officinale Rosc [Zingiberaceae; Zingiberis rhizoma Recens] (Sheng Jiang) and Areca catechu L [Arecaceae; Arecae pericarpium] (Da Fu Pi). Clinical observations have shown that Xinkang granules can significantly alleviate the clinical symptoms of patients with HFpEF, improve cardiac function (Li et al., 2013), reduce myocardial inflammatory cell infiltration (Liu et al., 2018), decrease cell apoptosis (Liu et al., 2019), inhibit ventricular remodeling (Liu et al., 2017), enhance myocardial contractility, increase the left ventricular ejection fraction, and reduce myocardial fibrosis. Further studies (Guo et al., 2023) have shown that Xinkang granules can inhibit the mRNA expression of NLRP3, caspase-1, and GSDMD, as well as the expression of NLRP3, caspase-1, and GSDMD-N, thereby affecting the activation and release of inflammatory factors and reducing the inflammatory response in HFpEF patients. Therefore, Xin Kang granules can improve the integrity of the myocardial cell membrane and ameliorate mitochondrial swelling in HFpEF model rats, indicating that this treatment significantly inhibits NLRP3 pathway-mediated pyroptosis.

#### 4.2.5 Fu Xin Tang

Fu Xin Tang is composed of botanical drugs such as Fu Zi and Ting Li Zi, which can treat HFpEF by improving heart function and reducing the level of NT-proBNP, thus enhancing the quality of life of patients. Experimental studies have shown (Zhang, 2022) that after treatment with Fu Xin Tang, the protein expression of NLRP3 in the cytoplasm of myocardial cells in mice with HFpEF decreases, as does the mRNA levels of NLRP3, ASC, and caspase-1 and the protein expression of NLRP3, ASC, pro-caspase-1, and cleaved caspase-1, indicating that Fu Xin Tang can inhibit the expression and activation of the NLRP3 inflammasome in mice with HFpEF. Additionally, the protein expression of Pro-IL-18, cleaved-IL-18, pro-IL-1β, and cleaved-IL-1β in HFpEF mice decreases after Fu Xin Tang treatment, suggesting that the preparation can inhibit the expression and activation of IL-1β and IL-18 and alleviate the inflammatory response in HFpEF. Fu Xin Tang can also inhibit the expression and activation of GSDMD and reduce pyroptosis in cardiomyocytes in HFpEF. Furthermore, Fu Xin Tang has multiple therapeutic effects in the treatment of HFpEF. Research (Duan, 2017) has shown that Fu Xin Tang may act on DAG transcription factors in cardiac fibroblasts, thereby inhibiting the DAG/PKC/ERK/NF-κB signaling pathway, affecting the activation and translocation of NF-κB, and inhibiting the expression of downstream inflammatory factors and pyroptosis, thus alleviating myocardial fibrosis, reversing ventricular remodeling, reducing the inflammatory response, and affecting the development of HFpEF. However, the specific mechanism by which Fu Xin Tang treats HFpEF still needs further study. The specific effective components and targets of Fu Xin Tang, as well as the pathways underlying its effects, still need further research. The mechanisms by which botanical drug preparations inhibit pyroptosis are listed in Table 2.

**TABLE 2:** Mechanisms by which botanical drug preparations inhibit pyroptosis.

Traditional Chinese medicine preparations	Component	Range tested	Model		Control		Treatment time	Mechanism and effect	Reference
					Control group	Experimental group			
Shenfu injection	Red ginseng, attached piece extract	6.0 mL/kg/d	Sprague-Dawley (SD) rats	The rat model of heart failure was prepared by subcutaneous multi-point injection of isoproterenol	Blank group; Model set	Shenfu injection group; MCC950 group	15 days	The expression levels of NLRP3, caspase-1, IL-1β, IL-18 and GSDMD were decreased. Inhibition of NLRP3 inflammasome activation	Fan et al. (2023)
Shenkui Tongmai granules	Radix astragali preparata, Cassia twig, Ganoderma lucidum, Epimedium, Salvia miltiorrhiza, Abelmoschus manihot, Poria cocos, Pericarpium citri reticulatae	0.72 g/kg/d; 1.44 g/kg/d; 2.88 g/kg/d	Sprague-Dawley (SD) rats	CHF rat model was prepared by epigrenal abdominal aortic coarctation	Sham group; Model set	Shenkui Tongmai granules low dose group; Shenkui Tongmai granules medium dose group; Shenkui Tongmai granules high dose group; Benazepril Group	4 weeks	Inhibiting the expression of TLR4 and NF-κB, inhibiting the production of caspase-1 and IL-1β precursor, and inhibiting the activation of caspase-1 and IL-1β	Yan et al. (2019)
Shengui-yixin soup	Astragalus, Radix pseudorrhoeae, Glycyrrhiza, Cassia twig, Salvia miltiorrhiza, Lepidium seed, Rhizoma alismatis, Safflower, Eupatorium, Pericarpium citri reticulatae	After soaking in water for 1 h, water decocting twice to extract a total of 300 m L juice, divided into two times in the morning and evening	Patients with Heart Failure	All of them met the relevant diagnostic criteria of the Revised Draft Guidelines for the Diagnosis and Treatment of Acute and Chronic heart Failure in Chinese Adults in 2013	Control group:Western medicine standard anti-heart failure drug treatment + Qiliqiangxin capsule	Treatment group:Western medicine standard anti-heart failure drug treatment + Shengui-yixin soup	4 weeks	It directly inhibited the protein expression of TLR4 in monocytes, inhibited the activation of NF-κB, blocked the phosphorylation of NF-κB and release into the nucleus, and regulated the transcription of inflammatory factors NF-α, IL-6, IL-1β	Feng et al. (2017)
Xinkang granule	Astragalus, Ginseng, Rattletop, Bupleurum, Platycodon grandiflorum, Poria cocos, Pericarpium citri reticulatae, Atractylodes macrocephala, Coix seed, Almond, Monkshood, Cassia twig, ginger peel, Pericarpium arecae	0.5 g/kg/d	Sprague-Dawley (SD) rats	Chronic heart failure model was prepared by intraperitoneal injection of Adr according to kg body weight	Normal group; Model set	Xinkang granules group; Valsartan Group	4 weeks	It can inhibit the mRNA expression of NLRP3, Caspase-1 and GSDMD in the classic pyroptosis pathway l, and inhibit the protein expression of NLRP3, Caspase-1 and GSDMD-N	Guo et al. (2023)
Fu Xin Tang	Aconite, Descurainia descuraini, Rhizoma alismatis, Xianling spleen, Angelica sinensis, Phellodendron Huangbai	3.80 mg/d; 7.58 mg/d; 15.16 mg/d	Wistar rats	Chronic heart failure model was prepared by intraperitoneal injection of Adr according to kg body weight	Western medicine group; Blank group	Fuxin decoction low; Medium and high dose group	2 weeks	It can inhibit the DAG/PKC/ERK/NF-κB signaling pathway and affect the activation and translocation of NF-κB, thereby inhibiting the expression of downstream inflammatory factors and the occurrence of pyroptosis	Duan. (2017)

## 5 Discussion

While studies of puerarin-targeted pyroptosis for the treatment of HF have conclusively demonstrated that puerarin has therapeutic potential for HF and attenuates the inflammatory response in rats with HF, the exact mechanisms linking the inflammatory response and HF, as well as the impact of inflammatory markers on the prognosis of HF, have not been explicitly addressed. In this article, the inflammatory response was shown to exacerbate myocardial fibrosis, ventricular remodeling, and HF by promoting pyroptosis, which in turn triggers chronic inflammation and tissue damage; the associated mechanisms of action are also discussed.

In in vitro and *in vivo* experiments of resveratrol, which targets pyroptosis for the treatment of HF, resveratrol targets and inhibits the activation of NLRP3 inflammatory vesicles, thereby reversing myocardial fibrosis, ventricular remodeling, and the decrease in cardiac function after myocardial infarction, thus preventing its progression to HF. However, notably, these functions were inconsistent in the results with different doses of resveratrol; thus, these discrepancies should be further investigated. In addition, previous results have also suggested that resveratrol has anti-aging effects, and such effects are also relevant for the treatment of HF; the link between its anti-aging effects and the inhibition of pyroptosis should also be investigated.

In the study of TP, which also targets pyroptosis for the treatment of HF, TP inhibited the expression of NLRP3 by inhibiting the activity of NF-κB, and TP also inhibited the interaction between NLRP3-ASC and the assembly of NLRP3 inflammatory vesicles, thus inhibiting pyroptosis and related inflammation, as well as collagen production; TP also alleviated myocardial fibrosis and improved cardiac function. Whether there is a deeper mechanism of interaction between TP and NF-κB and between TP and NLRP3-ASC needs to be further investigated. In this experiment, only a single dose was utilized and a concentration gradient to determine the optimal control concentration was not utilized; the use of a single dose is a shortcoming of this experiment. TP has a toxic effect, and its therapeutic concentration is close to the toxic concentration and even crosses over, so whether TP has toxic effects on other organs while treating HF needs to be considered. Furthermore, the relationship between its therapeutic efficacy and toxicology should be further studied.

Studies have shown that GRg1 has some therapeutic effects on myofibrosis in HF centers; previous studies have shown that GRg1 can treat myocardial fibrosis by inhibiting the TGFβ1-Smad pathway, and some studies have shown that it can play this role by inhibiting NLRP3-induced pyroptosis. Other previous studies have shown that in myocardial fibrosis, NLRP3-induced pyroptosis can act as an upstream response of the TGFβ1-Smad pathway. Therefore, whether GRg1 can exert an antimyocardial fibrosis effect by inhibiting pyroptosis and thus inhibiting the TGFβ1-Smad pathway is worthy of further investigation.

These results showed that DHM has certain therapeutic significance in DOX-induced congestive heart failure, and DHM could reduce the cardiotoxicity of DOX by upregulating SIRT1 expression and then targeting the inhibition of pyroptosis, but this mechanism needs to be confirmed by further *in vivo* experiments. Meanwhile, SIRT1 inhibition of pyroptosis also needs to be further explored.

All the experiments showed that colchicine could treat HF by targeting and inhibiting pyroptosis. However, none of the experiments involved were performed with NLRP3 knockout subjects. Whether colchicine directly alleviates HF by inhibiting the NLRP3 inflammasome and reducing pyroptosis remains unknown and requires further experimental verification.

The experiments consistently showed that Tanshinone IIA could target pyroptosis for the treatment of HF, but they also revealed a common problem with numerous experiments targeting pyroptosis for the treatment of HF, i.e., the potential mechanism of inhibition of pyroptosis by the plant metabolites needs to be further investigated in depth. Chai et al. made the suggestion that gene knockdown, overexpression, specific inhibitors, and other techniques could be used to further clarify the pathways that might be involved in targeting pyroptosis.

Experiments on Chinese medicinal preparations consistently show that the potential mechanism of action of agents in the treatment of HF is the inhibition of pyroptosis, and the treatment process and standardized control for each experiment also reflected the authenticity and validity of the data. However, the shortcomings are also clear, i.e., none of the experiments included experimental animals with knockdown of NLRP3 or pyroptosis-related inflammatory factors, and further experiments using the corresponding inhibitors were not performed. This shortcoming means that the direct link between the inhibition of pyroptosis by herbal preparations and their treatment of HF is unclear, and further experiments need to be performed. Meanwhile, the exact relationship between Chinese medicinal preparations and the classical pyroptosis pathway needs to be further studied.

## 6 Conclusion and prospects

This paper reviews the pathways involved in pyroptosis, and the most well-studied pathway, with main focus being the classic NLRP3/Caspase-1/GSDMD pathway. We discuss the associations among the key risk factors for HF, including the inflammatory response, myocardial hypertrophy, myocardial fibrosis, oxidative stress, and ionic disturbances. These risk factors often interact to promote the onset and progression of HF. In contrast, the pyroptosis pathway and the upstream PKC/ERK/NF-κB and TLR4/NF-κB pathways are potential targets for the treatment of HF. Although some progress has been made in the study of the classic pyroptosis pathway and its role in HF, studies of inflammasomes other than the NLRP3 inflammasome are lacking, and the upstream pathway also needs to be investigated.

TCM preparations have long been shown to be efficacious in treating HF. The discovery and study of pyroptosis mechanisms have provided a new theoretical basis for the treatment of HF with TCM formulations. In this paper, we review the ability of plant metabolites and botanical drug preparation to treat HF by inhibiting NLRP3, the NLRP3-mediated inflammatory response and pyroptosis, inhibiting its upstream pathway or both. Some plant metabolites, such as puerarin, dihydromyricetin, tanshinone IIA, and colchicine, have been experimentally reported to have certain therapeutic efficacy in treating HFpEF by targeting pyroptosis. Resveratrol and triptolide have been experimentally reported to have certain therapeutic effectiveness in treating HFrEF by targeting pyroptosis. Botanical drug preparations, such as Shenfu injection, Xinkang granules, and Fu Xin Tang, have been reported to target pyroptosis to treat HFpEF. Shenkui Tongmai granules and Shengui-Yixin soup have been reported to target pyroptosis to treat HFmrEF. This review also reveals some issues that need further research, including whether there is a special connection between different subtypes of heart failure and pyroptosis based on existing experimental reports on traditional Chinese medicine (TCM) extracts and botanical drug preparations. Targeting pyroptosis may also have therapeutic efficacy for other types of heart failure. Moreover, due to the lack of *in vitro* experimental validation, the specific pharmacological mechanism by which the plant metabolites described in this paper ameliorate and prevent HF through the inhibition of pyroptosis remains to be clarified. This is a priority for future research. Moreover, the NLRP3 signaling pathway and pyroptosis are expected to become new targets for the treatment of heart failure. The study of targeted inhibitors of pyroptosis is necessary, and the analysis of the metabolites of botanical preparations can also provide a reference for future studies. Moreover, more studies on the metabolites of botanical preparations as well as traditional preparations are needed.

The effectiveness of metabolites of TCM plants in the treatment of cardiovascular diseases is very important, and these metabolites have been widely used in China for the treatment of cardiovascular diseases with good clinical efficacy. It is particularly crucial to be able to prevent and treat HF, as the end stage of cardiovascular disease, via traditional Chinese medicine formulations. These formulations can not only delay the further development of cardiovascular diseases into HF but also improve the quality of life of patients with end-stage cardiovascular disease, enhancing their confidence in and enthusiasm for treatment. TCM focuses on the integration of nature and humanity, providing different treatment methods for different individuals. TCM preparations have been widely used in China and neighboring countries with beneficial effects. Considering the cultural beliefs surrounding TCM, we believe that the use of TCM formulations can be promoted in other countries, and further experiments and data are needed to achieve this in the future.

In clinical TCM, plant metabolites are used to treat HF with good efficacy, and as natural plant metabolites with fewer side effects, they are safer than chemically synthesized drugs. Therefore, TCM formulations are expected to be used in combination with other drugs for the treatment of HF to achieve greater clinical efficacy. However, due to the lack of a large amount of experimental data and objective evidence, the mechanism by which plant metabolites can treat HF is still unclear. Moreover, the side effects of these treatments need to be further studied. Botanical drugs are derived from ancient classic prescriptions or from self-prescribed prescriptions based on the years of clinical experience of TCM practitioners. These treatments are mostly composed of decoctions or raw material preparations of various Chinese medicines. The specific mechanism of action and side effects of botanical drug preparations used to treat HF need to be further examined. Therefore, research on the ability of TCM formulations to treat HF by targeting the pyroptosis pathway still is needed to overcome the following challenges.

First, the metabolites in botanical drug preparations are complex and have not been fully studied, and the therapeutic effects of these preparations on HF may be the result of the combined actions of multiple metabolites. The effect of botanical drug preparations on HF may not be limited to the inhibition of pyroptosis but may involve combined actions on multiple pathways. Although the specific mechanism underlying the effects of botanical drug preparation is difficult to determine, it is very important to do so. Second, patients with CHF need long-term treatment. Although plant metabolites and botanical drug preparations have few side effects, the safety of long-term treatment still needs to be considered; therefore, many studies evaluating clinical safety are necessary. Third, while there is a relatively large amount of animal research on the efficacy and safety of plant metabolites and botanical drug preparations, while research on clinical efficacy and safety is insufficient, and more clinical trials should be performed. Fourth, the study of the coordinated interactions between various plant metabolites and botanical drug preparations, including the use of several plant metabolites together to increase their efficacy or the combination of several plant metabolites to reduce toxic side effects, may be beneficial for the generation of new preparations. Fifth, among patients with HF, the prescriptions used often differ according to clinical symptoms. In clinical practice, patients with the same disease but different symptoms often does not respond well to the same prescription. Therefore, scientific studies on the effectiveness of TCM preparations is needed, and it is necessary to strengthen scientific research on TCM theory and diagnostic methods for the development of medicines.

In summary, pyroptosis contributes to the development and progression of HF in a variety of ways. Many experiments have shown that plant metabolites and botanical drug preparations that inhibit pyroptosis have certain advantages in the treatment of HF. It is necessary to further investigate the pathways related to pyroptosis, and the development of related inhibitors is a new direction for the treatment of HF.

## Figures and Tables

**Table:** 

**AHA**	American Heart Association
**Ang II**	Angiotensin Ⅱ
**ASC**	Apoptosis-associated speck-like protein containing a CARD
**AS-IV**	Astragaloside IV
**AT1**	Angiotensin type 1
**ATP**	Adenosine triphosphate
**Ca** ^**2+**^	Calcium ion
**CaMKⅡ**	Ca^2+^/calmodulin-dependent protein kinase Ⅱ
**CARD**	Caspase recruitment domain-containing protein
**CaSR**	Calcium-sensing receptor
**CFs**	Cardiac fibroblasts
**CHF**	Chronic heart failure
**DAG**	Diacylglycerol
**DAMPs**	Damage-related molecular patterns
**DCM**	Dilated cardiomyopathy
**DHM**	Dihydromyricetin
**DNA**	Deoxyribonucleic acid
**DOX**	Doxorubicin
**DrP1**	Dynamin-related protein 1
**dsDNA**	Double-stranded DNA
**ECM**	Extracellular matrix
**ER**	Endoplasmic reticulum
**ERK**	Extracellular signal-regulated kinase
**ERS**	Endoplasmic reticulum stress
**GLUT4**	Glucose transporter type 4
**GRg1**	Ginsenoside Rg1
**GSDMD**	Gasdermin D
**HF**	Heart failure
**HO-1**	Haem oxygenase 1
**IL**	Interleukin
**IP3**	Inositol triphosphate
**IRAK**	IL-1 receptor-associated kinase
**LPS**	Lipopolysaccharide
**LRRs**	Leucine-rich repeats
**LVEDD**	Left ventricular end-diastolic dimension
**LVEF**	Left ventricular ejection fraction
**LVESD**	Left ventricular end-systolic dimension
**LVFS**	Left ventricular fraction shortening
**LVMI**	Left ventricular mass index
**MAM**	Mitochondrial-associated membrane
**MANS**	Mitochondrial antiviral signaling protein
**MARK**	Mitogen-activated protein kinase
**MFN2**	Mitochondrial fusion protein 2
**MFs**	Myofibroblasts
**MMPs**	Matrix metalloproteinases
**mtDNA**	Mitochondrial DNA
**mtROS**	Mitochondrial ROS
**MyD88**	Myeloid differentiation primary response gene 88
**NACHT**	Nucleotide binding oligomerization domain
**NEK7**	NIMA-related kinase 7
**NF-κB**	Nuclear factor kappa-B
**NK**	Natural killer
**NLRC**	NLR family CARD domain-containing protein
**NLRP**	NLR family pyrin domain containing
**NLRs**	NOD-like receptors
**NOX**	Recombinant nicotinamide adenine nucleotide phosphate oxidase
**NT-proBNP**	N-terminal pro B type natriuretic peptide
**OMVs**	Outer membrane vesicles
**PAMPs**	Pathogen-related molecular patterns
**PARP-1**	Poly (ADP-ribose) polymerase 1
**PINK1**	PTEN-induced putative kinase 1
**PKC**	Protein kinase C
**PKR**	Protein kinase R
**PLC**	Phospholipase C protein
**PPAR**	Peroxisome proliferator-activated receptor
**PRRs**	Pattern recognition receptors
**PYD**	Pyrin domain
**RAGE**	Receptor of advanced glycation endproducts
**ROS**	Reactive oxygen species
**RyR2**	Ryanodine receptor 2
**SIRT1**	Sirtuin 1
**SOCE**	Store-operated Ca^2+^ entry
**SOD**	Superoxide dismutase
**STAT3**	Signal transducer and activator of transcription 3
**TAC**	Transverse aortic constriction
**TAK**	Transforming growth factor β-activated kinase
**TGF-β**	Transforming growth factor-beta
**TIMP**	Tissue inhibitor of metalloproteinase
**TLR4**	Toll-like receptor-4
**TLRs**	Toll-like receptors
**TNF**	Tumor necrosis factor
**TP**	Triptolide
**TUNEL**	Terminal deoxynucleotidyl transferase dUTP nick end labeling
**TXNIP**	Thioredoxin interacting/inhibiting protein
**VDAC**	Voltage-dependent anion channel
**VEGF**	Vascular endothelial growth factor
